# The Human Antibody Response to the Influenza Virus Neuraminidase Following Infection or Vaccination

**DOI:** 10.3390/vaccines9080846

**Published:** 2021-08-02

**Authors:** Madhusudan Rajendran, Florian Krammer, Meagan McMahon

**Affiliations:** 1 Department of Microbiology, Icahn School of Medicine at Mount Sinai, New York, NY 10029, USA; madhusudan.rajendran@gmail.com; 2 Department of Pathology, Icahn School of Medicine at Mount Sinai, New York, NY 10029, USA

**Keywords:** neuraminidase, antibodies, influenza

## Abstract

The influenza virus neuraminidase (NA) is primarily involved in the release of progeny viruses from infected cells—a critical role for virus replication. Compared to the immuno-dominant hemagglutinin, there are fewer NA subtypes, and NA experiences a slower rate of antigenic drift and reduced immune selection pressure. Furthermore, NA inhibiting antibodies prevent viral egress, thus preventing viral spread. Anti-NA immunity can lessen disease severity, reduce viral shedding, and decrease viral lung titers in humans and various animal models. As a result, there has been a concerted effort to investigate the possibilities of incorporating immunogenic forms of NA as a vaccine antigen in future vaccine formulations. In this review, we discuss NA-based immunity and describe several human NA-specific monoclonal antibodies (mAbs) that have a broad range of protection. We also review vaccine platforms that are investigating NA antigens in pre-clinical models and their potential use for next-generation influenza virus vaccines. The evidence presented here supports the inclusion of immunogenic NA in future influenza virus vaccines.

## 1. Introduction

**Figure 1:**
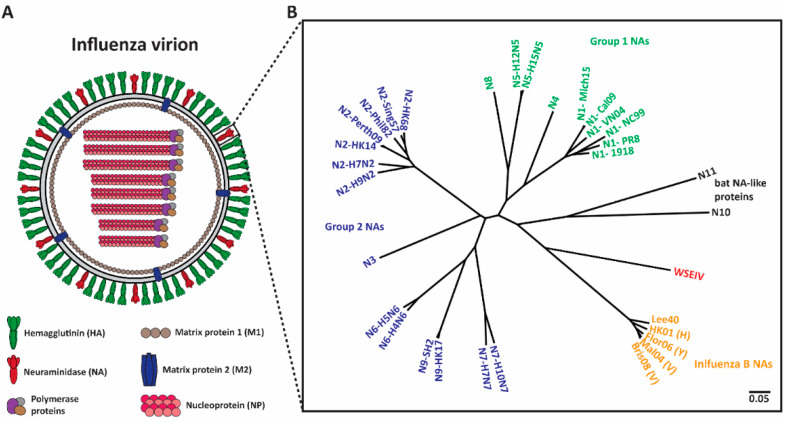
Phylogenetic tree of influenza NAs. (**A**) Depiction of an influenza virion. There are two major surface influenza glycoproteins: the hemagglutinin (HA) and neuraminidase (NA). (**B**) Phylogenetic tree of NA subtypes. Influenza A NAs comprise Group 1 (N1, N4, N5, and N8), Group 2 (N2, N3, N6, N7, and N9) and bat-like (N10 and N11) NAs. Influenza B NAs consist of Yamagata-like, Victoria-like and Hong Kong-like lineages. Wuhan spiny eel influenza virus (WSEIV) NA, a close relative of influenza B NAs, is also included in the phylogenetic tree. The scale bar represents a 5% change in amino acids. The phylogenetic tree was built using amino acids in Clustal Omega and then visualized in FigTree.

Vaccination remains the most effective countermeasure against influenza virus-associated morbidity and mortality [1,2,3,4]. Current seasonal influenza vaccines target the immuno-dominant surface glycoprotein, the hemagglutinin (HA) (Figure 1A) [2,5,6], as HA is responsible for viral attachment to sialic acid receptors on the host cell and fusion of viral and host endosomal membranes [6,7]. However, HA has high plasticity and changes constantly due to polymerase error rate and immune selection pressure, defined as antigenic drift [8]. As a result of this, seasonal vaccine strains must be updated annually, and, occasionally a mismatch between vaccine strains and circulating strains can result in seasonal epidemics [9,10,11]. Despite the necessity for the rapid production of seasonal influenza virus vaccines, the current process is time-consuming and expensive [12]. Hence, the investigation of new viral targets for influenza virus vaccines that are broadly protective, and do not change as frequently as HA, is warranted.

Neuraminidase (NA) (Figure 1A), the second surface glycoprotein of influenza virus, is a tetrameric type II transmembrane protein that plays several important roles in the viral replication cycle due to its enzymatic activity [13,14]. Initially, when an influenza virion enters a host, the virion needs to penetrate heavily glycosylated mucosal barriers [13,15,16]. These barriers act as decoy receptors for HA binding and neutralize the virion [13,17]. Here, NA assists the virion by releasing the virus particles from the decoy receptors, thus penetrating the mucus layer and gaining access to the underlying respiratory epithelium [13,15,16,17]. Upon entering and successfully replicating in the host cell, NA is crucial for viral detachment from the host cell by cleaving off sialic acid receptors that have adhered to HA [13,18,19]. Additionally, influenza virions are also known to adhere to each other via interactions between HA and sialic acids on glycans of other HAs, and between HA and other glycoproteins in the mucus layer [14,18]. NA prevents this aggregation and allows for the efficient spread of newly produced virions in the host and the subsequent transmission between hosts [14,20]. Interestingly, NA also plays a critical role in virus infectivity and HA-mediated membrane fusion [21]. 

Shifting the immune response towards the second major glycoprotein, NA, is a promising option for the improvement of seasonal vaccines. NA has a slower rate of antigenic drift, has fewer subtypes (Figure 1B), and lower immune selective pressure [22,23,24]. Hence, NA is an attractive target and anti-NA antibodies can inhibit the enzymatic activity of the virus via direct binding or steric hinderance of the active site [25]. Additionally, animal studies indicate that the induction of an anti-NA antibody response can confer protection [26,27,28]. Human challenge studies performed in the early 1970s revealed that anti-NA antibody titers inversely correlated with virus shedding and disease symptoms [29,30]. Recent studies indicate that NA inhibition (NI) titers independently correlated with protection against influenza virus symptoms and resulted in decreased viral shedding [31,32,33,34]. Understanding the role of anti-NA antibodies in controlling influenza virus infection can be improved through the generation of monoclonal antibodies (mAbs). In this review, we summarize several studies that isolated and characterized anti-NA antibodies from humans, and we discuss how this information will provide supporting evidence for the inclusion of standardized amounts of NA in future vaccine preparations.

### 1.1. NA-Based Immunity

Antibody responses towards influenza virus antigens typically target the two major surface glycoproteins, HA and NA (Figure 1A) [35]. Despite the importance of both anti-HA and anti-NA antibodies in preventing and controlling influenza virus infection, HA usually exhibits immunodominance over NA following influenza vaccination [13,36,37]. On the other hand, natural influenza virus infection induces more balanced antibody responses towards HA and NA [37]. Natural infection results in high seroconversion rates against both HA and NA, as measured by enzyme-linked immunosorbent assay (ELISA) [38,39]. A study in H1N1 pandemic influenza virus-infected patients demonstrated that seroconversion to NA could be observed at day 7 and peaked at day 28. However, NA antibodies began to decline by day 90 [39]. In the case of N2 antibodies, one study reported that N2 antibodies began to decline to undetectable levels within 5 months following infection, while another study reported persistence of detectable N2 antibodies up to 4 years after infection [40,41]. It should be noted that, in general, N1 antibody titers are lower than N2 and influenza B NA antibodies [42]. The lower titers of N1 antibodies might be caused by the lower immunogenicity of N1 but could also be an artifact of the reagents used to measure these antibody titers [38,39,42]. 

Several different types of influenza virus vaccines are currently in use to help protect against influenza virus infections. Immunoglobin responses towards NA after vaccination are substantially reduced when compared to infection [37]. Even though there are several different vaccines against influenza virus, only a handful of the vaccines can induce an immune response against NA, and several of the licensed vaccines contain little to no (e.g., Flucelvax) antigenic NA [43]. Live-attenuated virus vaccines (LAIV), whole inactivated influenza vaccine (IIV) and some split virus vaccines can induce NA antibody responses of varying degrees [34,44,45,46,47]. Similar to infection, antibodies in humans that developed post-vaccination peaked at 2–3 weeks; however, they only persisted for one year [48,49,50,51]. Additionally, route of administration can also have an effect on the humoral response against NA [52,53]. Unlike antibody responses to natural infection, antibody responses to vaccination are short-lived, and antibody titers induced by vaccination may even decline within a given influenza season [44,54,55]. NA-specific human monoclonal antibodies (mAbs) that are induced by natural infection and vaccination will be further discussed in the upcoming sections.

### 1.2. Human mAbs That Target NA

HA and NA-specific antibodies utilize different modes of action to control influenza virus infection. Anti-HA mAbs predominantly bind to the globular head domain and inhibit virus attachment and entry into the host cell [56,57]. Thus, HA-specific mAbs have potent neutralizing activity [58]. Additionally, some HA head-specific mAbs facilitate Fc receptor-mediated cytotoxicity, such as antibody dependent cellular toxicity (ADCC) [59,60]. Several studies have described human mAbs that are directed against the receptor binding site of HA, which have neutralizing activity and are broadly protective in mice [61,62,63,64]. In contrast to the head-specific mAbs, mAbs that bind to HA stalk inhibit viral-endosomal fusion [65]. Although the titers of stalk binding mAbs in humans are typically low, they bind to HA from different subtypes and have much broader neutralizing capacity and increased Fc-FcR activity when compared to mAbs targeting the head domain [5,65,66,67,68,69]. Different to anti-HA mAbs, anti-NA mAbs play a major role at the later stages of viral replication, specifically when the influenza virion buds off from the infected cells [18]. During the final stages of viral replication, NA enzymatically cleaves off sialic acid residues on the host cell surface, releasing virus progeny [18,19]. It is at this point that most of the anti-NA mAbs inhibit viral egress [13,70]. Since NA mAbs are mostly effective during viral egress, virus titer is not generally affected during infection in an in vitro plaque reduction assay [71,72,73,74]. However, the plaque diameter is significantly reduced in the presence of anti-NA mAbs [72,73,74]. Therefore, most of the mAbs against NA are non-neutralizing but are still able to inhibit the enzymatic activity of NA and prevent virion release and spread from the host cell [25]. Furthermore, some NA-specific mAbs also mediate ADCC, which in turn activates natural killer (NK) cells [20,75,76,77]. Upon activation via effector cells (e.g., NK cells, macrophages), they can produce the antiviral cytokine IFN-γ and degranulate or phagocytose infected cells, aiding in the clearance of virus-infected cells [60,77,78,79] 

**Figure 2:**
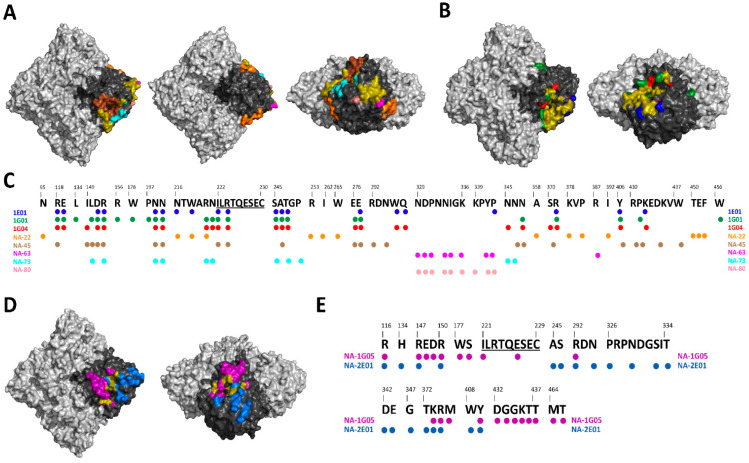
Mapping of NA-specific human monoclonal mAbs with known epitopes. (**A**) Top, bottom and side views of the A/Hunan/02650/2016 N9 (PDB ID: 6Q1Z) showing the epitopes of NA-22 in orange, NA-45 in brown, NA-63 in pink, NA-73 in teal, and NA-80 in salmon. (**B**) Top and side views of the A/California/04/2009 N1 (PDB ID: 6Q23) showing the epitopes of 1E01 in blue, 1G01 in green, and 1G04 in red. (**C**) Alignment of A/Hunan/02650/2016 N9 with the epitopes of 1E01, 1G01, 1G04, NA-22, NA-45, NA-63, NA-73, and NA-80. Universally conserved sequence “ILRTQESEC” is underlined. (**D**) Top and side views of the B/Perth/211/2001 NA (PDB ID: 3K38) showing the epitopes of NA-1G05 in purple and NA-2E01 in light blue. (**E**) Alignment of B/Perth/211/2001 with the epitopes of NA-1G05 and NA-2E01. Universally conserved sequence “ILRTQESEC” is underlined. For A, B and D overlapping epitopes between at least two mAbs are show in olive. Light gray denotes the NA tetramer, with the monomer highlighted in black.

Influenza virus vaccination and natural infection have the ability to induce a broad immune response against NA glycoprotein. This is demonstrated by the isolation of several human mAbs after both vaccination and natural infection. Even though some of the isolated human mAbs have a narrow reactivity, several of the isolated human mAbs have very broad reactivity spanning across both influenza A and influenza B strains (Figure 2 and Table 1). Below we describe human NA mAbs that have been isolated and their exciting reactivity.

#### 1.2.1. Group 1 and 2 mAbs

Natural infection with H1N1 and H3N2 induces a very high proportion of NA reactive B cells [27]. To assess the frequency of NA-reactive B cells activated during infection, Chen et al. characterized mAbs obtained from patients [37]. They isolated 128 influenza binding mAbs, with 15/88 being N1 reactive (from H1N1 infected patients) and 14/40 being N2 reactive (from H3N2 infected patients). 

Of the N1 reactive mAbs, 67% of them cross-reacted to the 1918 pandemic H1N1 strain, 33% reacted to various human H1N1 strains spanning the entire century, plus 20% bound to heterosubtypic strains. In vivo assessment of protection in mice indicated that all antibodies were protective in a prophylactic setting and four antibodies (EM-2E01, 1000–1D05, 1000-3B06 and 1000-3C05) were highly protective against challenge in a therapeutic setting when mice were challenged with A/Netherlands/602/2009. Of the N2 reactive mAbs, 86% reacted to the first pandemic H3N2 virus strain known to infect humans (A/Hong Kong/1/1968), 71% (10 of 14) of the antibodies reacted to the H2N2 influenza strain that circulated since 1957, eleven years prior, and 14% had cross-reactivity to heterosubtypic subtypes (N3 and N9). In vivo assessment of protection in mice indicated that eight of the N2 reactive antibodies were highly protective against challenge in both a therapeutic and prophylactic administration setting when mice were challenged with A/Philippines/2/1982 (H3N2).

Seasonal trivalent influenza vaccine (TIV) is also known to induce broadly reactive NA mAbs [47,80]. Human mAbs AG7C and AF9C were isolated from an individual vaccinated with the 2014–2015 Northern Hemisphere TIV. Even though both the mAbs were derived from the same individual, they showed significant sequence divergence. Both mAbs inhibited NA spanning over 80 years, with AG7C inhibiting N1 from A/Brevig Mission/1/1918 [80]. Additionally, when administered to mice, AG7C did not require Fc engagement for complete protection, indicating various modes of protection elicited by NA mAbs [80].

Pandemic preparedness necessitates the assessment of anti-NA antibody responses against avian influenza viruses. One such study by Gilchuk et al. isolated human N9 mAbs following a A/Shanghai/2/2013 H7N9 monovalent IIV vaccination or A/British Columbia/1/2015 H7N9 natural infection [70]. Similar to other avian NAs, N9 NA has two functional sites: the sialidase enzyme site and hemadsorption site [18]. None of the isolated anti-N9 mAbs bound to the hemadsorption site, only bound to the sialidase enzymatic site. Out of the 35 isolated human mAbs, only a handful were characterized in detail: NA-22, NA-45, NA-63, NA-73, NA-80 and NA-97 (Figure 2A,C). NA-97 was one of the mAbs isolated after a H7N9 natural infection, while NA-22, NA-45, NA-63, NA-73 and NA-80 were all isolated post-H7N9 vaccination. Almost all of the isolated mAbs were subtype specific except for NA-97, which cross-reacted with N6 [70]. Three of the five mAbs, NA-22, NA-63, and NA-80, inhibited NA enzyme activity via steric hindrance, preventing NA binding to the sialic acid site, while NA-45 and NA-73 inhibited the enzymatic activity via direct binding to the enzymatic site [70,81,82]. Similar results were observed in a sialoside glycan array assay when A/Shanghai/2/2013 N9 was incubated in the presence of one of the mAbs (NA-22, NA-45, NA-63, NA-73 or NA-80) [81]. Therefore, the anti-N9 mAbs neutralize the H7N9 virus primarily by steric hindrance of NA active site, which results in the egress inhibition of progeny virions. The isolated human mAbs also completely protected mice prophylactically (NA-22, NA-45, NA-73 and NA-80) and therapeutically (NA-73 and NA-80) [70,82]. Interestingly NA-22, a very weak neuraminidase inhibition (NAI) antibody was still able to completely protect mice after a H7N9 virus challenge. Upon further characterization, it was noted that NA-22 utilized Fc-mediated effector function to protect mice against H7N9 infection [70]. When investigated in detail, it was concluded that NA mAbs usually bind to three general epitope regions [70,81,82]. Antibody NA-45 directly binds to the enzymatic site with partial sialic acid mimicry (Figure 2A,C) [81,82]. Unique to NA-45, the mAb is able to encompass the whole NA active site [81]. NA-63, NA-73 and NA-80 all bound to epitopes proximal to the active site, with NA-73 binding to a conformational epitope, and NA-63 and NA-80 binding to linear epitopes (Figure 2A,C) [81,82]. Lastly, NA-22 binds to an epitope at the protomer interphase (Figure 2A,C) [81,82]. Interestingly, even though NA-73 bound to epitopes proximal from the active site, it can still inhibit NA activity when the small substrate, NANA, is used [70,81,82]. A possible explanation might be that NA-73 binding to the epitope proximal to the active site induces a slight allosteric change in the NA active site, making the cleaving of smaller substrates impossible.

#### 1.2.2. Influenza B mAbs

Studies observing human mAbs isolated after influenza B virus infection are also beginning to emerge. Madsen et al. isolated seven different human mAbs from an individual infected with influenza virus [83]. Of the seven antibodies, six (NA-1A03, NA-1G05, NA-2D10, NA-2E01, NA-2H09 and NA-3C01) showed broad reactivity against influenza B virus strains spanning over more than 70 years of antigenic drift, going back as far as the ancestral strain B/Lee/1940. Assessment of NA inhibition indicated that five out of seven mAbs (NA-1G05, NA-2D10, NA-2E01, NA-2H09 and NA-3C01) had broad inhibition of influenza B viruses, inhibiting as far back as the ancestral B/Lee/1940 strain. Further determination of active site inhibition using the NA-Star assay suggested that NA-1G05 and NA-2E01 mAbs are able to bind to the active site of the NA enzyme. This result was confirmed using single particle cryo-electron microscopy of either NA-1G05 or NA-2E01 in complex with B NA, indicating that both the mAbs target the active site, with the CDR-H3 loops from both NA-1G05 and NA-2E01 binding similarly to the NA inhibitor, oseltamivir (Figure 2D,E). Importantly, NA-1G05 and NA-2E01 were also shown to be broadly protective in vivo when mice were challenged with a B/Yamagata/16/88-like or B/Victoria/2/87-like mouse adapted stains from the 2017–2018 and 2018–2019 influenza seasons, respectively. 

Piepenbrink et al. isolated NA human mAbs from individuals who were vaccinated with a quadrivalent inactivated influenza vaccine. The authors were able to isolate broadly reactive mAbs against influenza B virus NA. Some of the isolated mAbs (1086C12, 1092D4, 1092E10, 1122C7) recognized the common ancestor B/Lee/1940 [84]. The authors identified members of the 1092D4, 1092E10 and 1122C7 clonal lineage one year after vaccination, indicating that influenza B NA-specific B cell lineage with protective potential remaining within the CD138+ bone marrow plasma cell repertoire following inactivated influenza vaccination [84]. 

#### 1.2.3. Pan NA mAbs

Despite their broad within-group binding, the anti-NA mAbs described so far were not found to bind cross-group. An exciting development relevant to this point is the recent paper published by Stadlbauer et al. [85]. Here, the authors isolated and characterized three broadly binding NA mAbs (1G04, 1E01 and 1G01) from a H3N2 infected patient (Figure 2B,C). These mAbs were found to have long complementarity determining regions’ H3 domains, which allowed antibody binding deep within the NA active site. NA binding and inhibition characterization of these mAbs found that they were broadly reactive to and inhibiting of group 1, group 2 and influenza B NAs (Figure 2B,C). Further characterization of the mAbs in an in vivo setting indicated that administration of these mAbs prior to challenge lead to broad-cross protection of both group 1, group 2 and influenza B viruses, with 1G01 being protective against every challenge virus tested [85]. Interestingly, 1G01 was also found to bind to the NA from a Wuhan spiny eel influenza virus, a virus isolated from the gill tissues of lesser spiny eels [86].

### 1.3. NA Human mAbs Inform Vaccine Design

The development of NA vaccine antigens is complicated by several factors. The skewed antibody response towards HA is mainly due the presence of approximately four times more HA than the NA on the influenza virion surface [87]. As a result of the immunodominance of HA over NA, HAs evolve more quickly than NAs. A H3N2 virus study showed that the globular domain of HA evolves at a rate of 12.9 × 10^−3^–14.9 × 10^−3^ amino acid/site/year compared to NA, which evolves at a rate of 9.1 × 10^−3^ amino acid/site/year [56,88]. While antibody responses against NA are the primary drivers of the antigenic drift, antibody response and altered affinity for NA/HA receptors play a role in NA/HA antigenic drift [33,89]. Furthermore, immunization with the same amount of purified HA and NA resulted in similar increases in antibody titers to each of the antigens, demonstrating that the two antigens have very similar immunogenicity [90]. Due to the lower drift and immunogenic properties of NA, there has been a concerted effort to use NA as a vaccine antigen [20,46,88].

As discussed in the above section, several broadly reactive human NA mAbs have been isolated either after natural infection or post-vaccination. These human NA mAbs display a broad range of protection ranging from homologous protection to different influenza subtypes. For example, human mAbs NA-22, NA-45, NA-63, NA-73 and NA-80 are only active against N9 subtypes [70,81] (Figure 2A). NA-1G05 and NA-2E01 are reactive against all influenza B types [83] (Figure 2D). Lastly, 1E01 and 1G01 are broadly reactive against all influenza A and B types [85] (Figure 2B). The identification of broadly reactive mAbs indicates the presence of conserved epitopes on NA antigen which can be utilized for future NA vaccine candidates (Figure 2C,E). Interestingly, children born after 2006 showed ELISA antibody titers against the ancestral A/South Carolina/1/1918 and B/Lee/1940 influenza virus strains. The ELISA antibody titers correlated positively with NAI titers [42]. Additionally, a recent clinical study in which healthy young adults were challenged with pandemic H1N1 demonstrated differences in the role of HA and NA-specific antibodies. While reduction in virus shedding correlated with HA inhibition titers; fewer symptoms, reduced symptom severity score, reduced duration of symptoms and reduced viral shedding correlated with NAI titers [31]. It has also been shown that NAI titers are independent predictors of immunity against the influenza virus and are an independent correlate of protection [33,34]. These protective mAbs against NA have three different mechanisms of inhibition: (i) direct inhibition of NA catalytic site, (ii) indirect inhibition of NA catalytic site via steric hindrance, and (iii) mAb with little to no NAI activity utilize Fc-FcR-based effector functions [75,85].

Antibodies against NA are not directly involved with preventing virus binding to the host receptors, similar to some anti-HA antibodies. Thus, anti-NA mAbs are not expected to inhibit infection but limit viral spread within the host, reduce morbidity and mortality, decrease viral shedding and reduce transmission to naïve hosts [90,91]. Thus, vaccines containing immunogenic amounts of both HA and NA would be optimal to provide complete protection against influenza virus infection [92]. HA and NA ratios are different for different subtypes and different strains within a subtype [93]. Therefore, NA content and HA:NA ratio in future vaccine candidates need to be standardized. Different assays such as mass spectrometry (MS), isotype dilution MS and capture ELISA to measure the potency of NA in vaccine preparations are under development [93,94,95]. Induction of broadly cross-reactive mAbs has indicated that NA is immunogenic, and that NA antigen contains broadly conserved epitopes. 

These studies demonstrate the growing potential of using NA as a vaccine antigen. Advances in emerging platforms (discussed below), a greater understanding of NA structural biology and mAb characterization can inform the design and development of NA vaccine antigens that promote a broad antibody response. Even though the different studies discussed here provide evidence for the use of NA as a vaccine antigen, a slew of questions remain unanswered. The factors that drive long-lasting NA-specific immunity are not well understood. This knowledge could be beneficial in designing NA-based vaccines. What makes natural infection provide a broader and long-lasting antibody response compared to vaccination? Testing of the novel vaccine platforms that use NA as the primary antigen have, so far, been mostly restricted to mice, with only limited platforms assessed in guinea pigs and ferrets (Table 2). Therefore, could a NA vaccine platform that induces robust immune response in mice perform similarly in ferrets and guinea pigs? None of the currently licensed vaccines have standardized amounts of NA. In future vaccine preparations, should NA antigens be standardized to similar amounts or greater amounts than HA to produce a robust immune response? Current studies have shown that NA antigenically drifts at a much slower rate compared to HA. How will the development of a vaccine targeting NA potentially influence the evolution rate of NA? In addition, newly developed assays such as MS, isotype dilution MS and capture ELISA to measure potency of NA in vaccine preparations have been great tools in propelling NA as a vaccine antigen in future vaccine preparations [93,94,95]. Future studies that try to answer the above-mentioned questions along with several others are vital in the development of a future NA-based vaccines.

### 1.4. Emerging Platforms for the Development of NA-Based Vaccines

Vaccine candidates that target NA have been frequently revisited since the 1968 Hong Kong influenza A (H3N2) pandemic. The first NA-based inactivated vaccine, which consisted of an irrelevant equine HA and a NA from A/Hong Kong/1/1968 (H3N2), protected against challenge with a virus carrying an antigenically identical NA but a mismatched HA [29]. Despite these encouraging results, NA as a vaccine antigen has only received limited attention in the past. Early immunogenicity studies did not frequently evaluate antibody responses against NA as it was difficult to perform the assay safely, reproducibly and at high throughput [111,112,113]. Furthermore, the amount of NA varied in different viruses and was not easily quantified [20]. Lastly the unstable nature of NAs resulted in conflicting immunogenicity studies [111,114]. As a result, the development of NA-based vaccines using traditional egg-based vaccine platforms has been relatively inactive since 1998 [114]. Emerging vaccine platforms, such as modified inactivated vaccines, recombinant NAs, virus-like particles (VLP), virus replicon particles (VRP), viral vector platforms and nucleic acid vaccines (Table 2), could be used to overcome previously unsuccessful attempts to develop NA as a vaccine antigen. Here we will describe these vaccine platforms and how they have been used in a pre-clinical setting to induce NA antibody responses. 

#### 1.4.1. Modified Inactivated Vaccines

IIVs contain both HA and NA; however, IIVs are only standardized to the amount of HA [115]. Regardless, IIVs still contain immunogenic amounts of NA [46]. A preliminary study of monovalent and trivalent seasonal IIVs and split trivalent influenza vaccines suggested that NAs remain active over the vaccine shelf-life [116]. However, the stability of NAs in IIVs is subtype dependent. Analysis of IIV preparations indicates that (i) group 2 NAs are more thermostable than group 1 and influenza B NAs, (ii) influenza B NAs are the most resistant to detergent treatment, and (iii) group 1 NAs are the most resistant of to freeze–thaw cycles [116]. Even though IIVs are not standardized to the amount of NA, immunogenicity against NA can be increased by extending NA stalk domain via insertion of several amino acids. Mice immunized with IIVs containing A/Puerto Rico/8/1934 N1 with 30 amino acid extended stalk domain induced significantly higher anti-NA antibodies than mice immunized with wild type NA. Interestingly, the extension of NA stalk domain did not affect antibody levels against HA. Similar results were observed when mice were immunized with A/Hong Kong/5738/2014 N2 that had a 15 amino acid insertion [96]. In an interesting study by Zheng et al., swapping the 5′ and 3′ terminal packaging signals of the A/Puerto Rico/8/1934 NA led to increased anti-NA antibodies in mice vaccinated with the rewired NA when compared to mice that were vaccinated with unmodified viruses [117]. In order to understand if the extension of NA of stalk or if rewiring RNA packaging signals can induce a broader immune response against different subtypes, future studies that compare the protective effects of the extended NA stalk IIV against different influenza virus subtypes are warranted.

#### 1.4.2. Recombinant NA Vaccines

Recombinant NA vaccines only contain the purified recombinant NA against which immune responses are directed. In human trials, purified recombinant A/Beijing/32/1993 N2-based vaccines were shown to be safe and produced four-fold seroconversion at doses ≥7.7 µg in healthy adults, compared to baseline sera [97]. Wohlbold et al. found that mice vaccinated with recombinant NA, purified from baculovirus-infected insect cell system, were protected against homologous and heterologous influenza virus infection. Passive transfer of sera from vaccinated mice to naïve mice protected naive mice from challenge, indicating that humoral immunity is sufficient for protection [27]. Interestingly, guinea pigs vaccinated with recombinant B/Malaysia/2506/2004 NA intranasally showed reduced virus titers, and vaccination fully prevented homologous transmission from vaccinated donors to naïve recipients [102]. Computationally engineered recombinant NA antigens, NA5200, NA7900 and NA9100, were designed based on sequence clusters encompassing three major groupings of N1 sequence space. Of note, NA7900 protected against all seasonal H1N1 viruses tested, and NA9100 showed the broadest range of protection covering N1s spanning more than 85 years [99]. Lastly, when comparing the efficacy of conventional IIVs, LAIVs and recombinant NA-based vaccine in a murine model, it was found that, irrespective of influenza A or B viruses, only recombinant NA-based vaccine protected mice against challenge with heterologous virus strains, inducing a greater than two-fold increase in NAI titers compared to the PBS vaccinated animals [100,101]. Due to the efficacy and broad protection against influenza viruses following vaccination with recombinant NA vaccines, this vaccine platform should be further explored.

#### 1.4.3. Virus Like Particles (VLPs)

VLPs are multiprotein structures that mimic the conformational, structural and antigenic properties of authentic native viruses, but lack the complete viral genome, potentially yielding a safer and cheaper vaccine [118]. VLPs can imitate the antigenic properties of influenza viruses, making them ideal candidates for the development of NA-based vaccines [118,119]. Ferrets vaccinated with a VLP vaccine composed of A/Indonesia/05/2005 N1 were protected from lethal H5N1 challenge, elicited higher NAI antibody titers and shed less infectious viruses compared to similarly challenged control animals that did not receive the VLP vaccine [28]. Heterologous protection in mice vaccinated with N1 VLPs has also been observed. Mice immunized with VLP expressing pandemic N1 were completely protected against infection from a homologous virus and H5N1 infection [104]. It should be noted that several prophylactic VLP-based vaccines are already licensed for hepatitis B virus and human papillomavirus [120]; however, the development of VLP-based influenza vaccines may be complicated by the lack of a self-assembling capsid and baculovirus contaminants. 

#### 1.4.4. Viral Replicon Particles (VRPs)

Single-stranded RNA viruses of both positive and negative polarity have been used as vectors for vaccine development [121]. VRPs are self-amplifying RNAs that are avirulent and are unable to revert to virulence [122]. Halbherr et al. characterized protective properties of mono-specific immune sera that were generated by vaccination with VRP encoding A/swine/Belzig/2/2001 N1 and A/swan/Potsdam/62/81 N7 [105]. The immune sera inhibited hemagglutination in an NA subtype specific and HA subtype independent manner, interfered with infection of Madin–Darby canine kidney cells, and inhibited enzymatic activity of a number of NA subtypes. Furthermore, chickens immunized with VRPs encoding A/chicken/Yamaguchi/7/2004 N1, and then infected with low pathogenic avian influenza virus showed significantly reduced inflammatory serum markers and complete elimination of virus shedding [105]. Studies that use VRPs containing NA as the vaccine antigen are limited. Therefore, further research in different hosts and testing the effectiveness of VRP-based NA vaccines against heterologous influenza strains are needed. 

#### 1.4.5. Viral Vector Vaccines

Replication incompetent viral vectors, with the ability to induce both humoral and cell-mediated immune responses, are also being evaluated for use as NA-based vaccines [123,124,125]. The viral vectors are non-infectious to the host but can express the antigen over a certain period of time [125]. Mice vaccinated with a parainfluenza virus 5 (PIV5) viral vector expressing either an avian N1 or a pandemic N1 elicited a robust NA-specific antibody response in mice. These mice were protected against both homologous and heterologous influenza virus challenge [106]. Similarly, mice vaccinated with modified vaccinia virus Ankara (MVA) vectors expressing N3 and N9 antigens had high levels of N3 and N9-specific antibodies. Furthermore, mice immunized with MVA-N3 vector were protected against A/mallard/Netherlands/12/2000 H7N3 virus challenge, and partially protected against A/Shanghai/02/2013 H7N9 virus challenge [107]. These studies suggest that viral vector vaccine platforms may prove to be very useful for NA-based vaccine development.

#### 1.4.6. DNA Vaccines

Developed two decades ago, DNA vaccines are non-infectious, non-replicating, and do not induce vector-specific immunity, making them attractive for vaccine development [126]. Mice immunized with a A/Puerto Rico/8/1934 N1-DNA vaccine have complete protection against a homologous virus challenge and partial protection against heterologous challenge [108]. In support, mice were administered A/Aichi/2/1968 N2-DNA vaccine and then challenged with lethal doses of homologous or heterologous viruses. The N2-DNA vaccine protected mice against infection with homologous viruses, and drifted viruses by inducing a greater than two-fold increase in NAI titers. However, the N2-DNA vaccine failed to protect infection by H1N1 influenza virus [109]. Promising approaches have arisen from numerous studies evaluating different DNA vaccine formulations and delivery systems, making DNA vaccine technology a reliable platform for NA-based vaccine formulation.

#### 1.4.7. RNA Vaccines

RNA-based vaccines are the most recent version of the nucleic acid-based vaccines and possess several benefits over DNA vaccines. In the early 1990s, it was already demonstrated that direct injection of the messenger RNA (mRNA) in the mouse model, resulted in the expression of the encoded protein [127]. Compared to DNA vaccines, which function by the DNA entering the nucleus, mRNA vaccines function by the translation of mRNA in the cytoplasm [128,129]. Freyn et al. used a nucleoside modified mRNA influenza vaccine with multiple antigens, mini-HA (HA stalk domain alone), NA, M2 and NP, in order to observe the protective efficacy of such a vaccine in mice [110]. Of all the mRNA antigens they tested, vaccination with A/Michigan/45/2015 N1-mRNA out-competed all other components when the mice were challenged with a pandemic H1N1 strain. Interestingly, injection with N1-mRNA produced antibodies that protected mice up to a challenge dose of 500 times the 50% lethal dose. Notably, the N1-mRNA dose could be reduced to as low as 0.05 µg of mRNA and mice were still protected against the H1N1 challenge [110]. Even though studies using NA-based mRNA vaccine platform are very limited, the NA-mRNA vaccine platform seems promising and should be investigated further. 

As we describe above, emerging vaccine platforms that utilize NA as a primary antigen have potential for being incorporated. As each vaccine platform is at a different stage of development and offers varying breadths of protection, it may be hard to address the full potential of any one vaccine platform. Despite this, most vaccine platforms indicate that NA is a suitable antigen for incorporation into these vaccine platforms.

## 2. Conclusions

Here, we described the human antibody response to NA, the immuno-subdominant glycoprotein found on the surface of the influenza virion. We also discussed emerging vaccine platforms that have the potential to target the NA, thereby inducing NA-specific antibody responses. We believe the NA to be a fascinating protein that plays multiple essential roles in the influenza virus life cycle, that by targeting, would lead to increased protection when compared to current influenza vaccines that target only the immunodominant HA. Emerging vaccine platforms represent a more attractive target in this regard, as current vaccines are standardized to the amount of HA. As such, targeting this antigen with emerging platforms would be beneficial to human health as NA could be given at equal amounts to the HA. In order to confirm that role and to harness NA-based immunity optimally to enhance the breadth of influenza virus vaccines and increase vaccine efficacy, further characterization and understanding of mAbs that bind NA will help inform next generation influenza virus vaccines, allowing the full potential of NA as a vaccine antigen to emerge.

## Figures and Tables

**Table 1:** Summary of NA mAbs isolated from humans.

Reactivity	Ref.	mAb Name	Induced after
Group 1 NA	[37]	1000-3C05, 1000-2E06, 1000-3B04, 1000-3B06, EM-2E01, 1000-1D05, 1000-1E02, 1000-1H01, 294-G-1F01, 294-A-1C02, 295-G-2F04, 300-G-2A04, 300-G-2F04, 294-A-1C06	H1N1 infection
	[80]	AG7C, AF9C	Seasonal trivalent inactivated vaccine
Group 2 NA	[37]	229-1D05, 235-1C02, 235-1E06, 294-1A02, 228-1B03, 228-3F04, 2291B05, 229-1F06, 229-1G03, 229-2B04, 229-2C06, 229-2E02	H3N2 infection
	[70,81,82]	NA-97	A/British Columbia/1/2015 (H7N9) natural infection
	[70,81,82]	NA-22, NA-45, NA-63, NA-73, NA-80	A/Shanghai/2/2013 (H7N9) monovalent inactivated influenza vaccine
Influenza B NA	[83]	NA-1A03, NA-1G05, NA-2D10, NA-2E01, NA-2H09, NA-3C01	Influenza B infection
	[84]	1086C12, 1092D4, 1092E10, 1122C7	Quadrivalent inactivated influenza vaccine
Pan NA		1G01, 1E01, 1G04	H3N2 infection

**Table 2:** Summary of emerging NA-based vaccine platforms against influenza viruses described in this review. + indicates low immunogenicity, +++ indicates high immunogenicity, N.D. indicates not determined. AA indicates amino acid.

Platform	NA Antigen Subtype	Animal Model	Immunogenicity	Protection	Ref.
Inactivated vaccine	30 AA insertion in seasonal N1 15 AA insertion in N2	Mice	+++ +++	N.D.	[96]
Recombinant NA vaccine	N2	Human	+	N.D.	[97]
	Seasonal N1 N2 B/Yamagata/16/88-like B-NA	Mice	+++ +++ +++	Homologous Heterologous	[27]
	Avian N1 Pandemic N1	Mice	+	Homologous Partial heterologous	[98]
	N1	Mice	+++	Homologous	[99]
	N2	Mice	+++	Homologous Partial heterologous	[100]
	B-NA	Mice Guinea pigs	+	Homologous Heterologous	[101]
	B-NA	Guinea pigs	+++	Homologous Partial heterologous	[102]
Virus like particles	Avian N1	Ferrets	+++	Homologous	[28]
	Pandemic N1	Mice	+	Homologous Heterologous	[103]
	Avian N1 Seasonal N1	Mice	+++	Homologous Heterologous	[104]
Viral replicon particles	Avian N1	Chicken	+++	N.D.	[105]
Viral Vector vaccines	Avian N1 Pandemic N1	Mice	+++	Homologous Heterologous Heterosubtypic	[106]
	N3 N9	Mice	+++	Homologous	[107]
Nucleic Acid-DNA	Seasonal N1	Mice	+	Homologous Partial heterologous	[108]
	N2	Mice	+	Homologous Partial heterologous	[109]
Nucleic Acid-RNA	Seasonal N1	Mice	+++	Homologous	[110]

## References

[1] Bernstein D.I., Guptill J., Naficy A., Nachbagauer R., Berlanda-Scorza F., Feser J., Wilson P.C., Solorzano A., Van der Wielen M., Walter E.B. (2020). Immunogenicity of chimeric haemagglutinin-based, universal influenza virus vaccine candidates: Interim results of a randomised, placebo-controlled, phase 1 clinical trial. Lancet Infect. Dis..

[2] CDC (2020). Vaccine Effectiveness: How Well Do the Flu Vaccines Work?. https://www.cdc.gov/flu/vaccines-work/vaccineeffect.htm.

[3] CDC (2018). Who Is at High Risk for Flu Complications. https://www.cdc.gov/flu/highrisk/index.htm.

[4] WHO (2018). Influenza (Seasonal). https://www.who.int/en/news-room/fact-sheets/detail/influenza-(seasonal).

[5] Nachbagauer R., Liu W.C., Choi A., Wohlbold T.J., Atlas T., Rajendran M., Solorzano A., Berlanda-Scorza F., Garcia-Sastre A., Palese P. (2017). A universal influenza virus vaccine candidate confers protection against pandemic H1N1 infection in preclinical ferret studies. NPJ Vaccines.

[6] Shaw M.L., Palese P., Knipe D.M., Howley P.M. (2013). Orthomyxoviridae: The Viruses and Their Replication. Fields Virology.

[7] Bouvier N.M., Palese P. (2008). The Biology of Influenza Viruses. Vaccine.

[8] Heaton N.S., Sachs D., Chen C.J., Hai R., Palese P. (2013). Genome-wide mutagenesis of influenza virus reveals unique plasticity of the hemagglutinin and NS1 proteins. Proc. Natl. Acad. Sci. USA.

[9] Xie H., Wan X.F., Ye Z., Plant E.P., Zhao Y., Xu Y., Li X., Finch C., Zhao N., Kawano T. (2015). H3N2 Mismatch of 2014-15 Northern Hemisphere Influenza Vaccines and Head-to-head Comparison between Human and Ferret Antisera derived Antigenic Maps. Sci. Rep..

[10] De Jong J.C., Beyer W.E., Palache A.M., Rimmelzwaan G.F., Osterhaus A.D. (2000). Mismatch between the 1997/1998 influenza vaccine and the major epidemic A(H3N2) virus strain as the cause of an inadequate vaccine-induced antibody response to this strain in the elderly. J. Med. Virol..

[11] CDC (2018). Past Pandemics. https://www.cdc.gov/flu/pandemic-resources/basics/past-pandemics.html.

[12] Krammer F., Palese P. (2015). Advances in the development of influenza virus vaccines. Nat. Rev. Drug. Discov..

[13] Wohlbold T.J., Krammer F. (2014). In the shadow of hemagglutinin: A growing interest in influenza viral neuraminidase and its role as a vaccine antigen. Viruses.

[14] Eichelberger M.C., Wan H. (2015). Influenza neuraminidase as a vaccine antigen. Curr. Top. Microbiol. Immunol..

[15] Matrosovich M.N., Matrosovich T.Y., Gray T., Roberts N.A., Klenk H.D. (2004). Neuraminidase is important for the initiation of influenza virus infection in human airway epithelium. J. Virol..

[16] Cohen M., Zhang X.Q., Senaati H.P., Chen H.W., Varki N.M., Schooley R.T., Gagneux P. (2013). Influenza A penetrates host mucus by cleaving sialic acids with neuraminidase. Virol. J..

[17] Ma J., Rubin B.K., Voynow J.A. (2018). Mucins, Mucus, and Goblet Cells. Chest.

[18] McAuley J.L., Gilbertson B.P., Trifkovic S., Brown L.E., McKimm-Breschkin J.L. (2019). Influenza Virus Neuraminidase Structure and Functions. Front. Microbiol..

[19] Shtyrya Y.A., Mochalova L.V., Bovin N.V. (2009). Influenza virus neuraminidase: Structure and function. Acta Nat..

[20] Krammer F., Fouchier R.A.M., Eichelberger M.C., Webby R.J., Shaw-Saliba K., Wan H., Wilson P.C., Compans R.W., Skountzou I., Monto A.S. (2018). NAction! How Can Neuraminidase-Based Immunity Contribute to Better Influenza Virus Vaccines?. mBio.

[21] Su B., Wurtzer S., Rameix-Welti M.A., Dwyer D., van der Werf S., Naffakh N., Clavel F., Labrosse B. (2009). Enhancement of the influenza A hemagglutinin (HA)-mediated cell-cell fusion and virus entry by the viral neuraminidase (NA). PLoS ONE.

[22] Kilbourne E.D., Johansson B.E., Grajower B. (1990). Independent and disparate evolution in nature of influenza A virus hemagglutinin and neuraminidase glycoproteins. Proc. Natl. Acad. Sci. USA.

[23] Westgeest K.B., de Graaf M., Fourment M., Bestebroer T.M., van Beek R., Spronken M.I., de Jong J.C., Rimmelzwaan G.F., Russell C.A., Osterhaus A.D. (2012). Genetic evolution of the neuraminidase of influenza A (H3N2) viruses from 1968 to 2009 and its correspondence to haemagglutinin evolution. J. Gen. Virol..

[24] Schulman J.L., Kilbourne E.D. (1969). Independent variation in nature of hemagglutinin and neuraminidase antigens of influenza virus: Distinctiveness of hemagglutinin antigen of Hong Kong-68 virus. Proc. Natl. Acad. Sci. USA.

[25] Couzens L., Gao J., Westgeest K., Sandbulte M., Lugovtsev V., Fouchier R., Eichelberger M. (2014). An optimized enzyme-linked lectin assay to measure influenza A virus neuraminidase inhibition antibody titers in human sera. J. Virol. Methods.

[26] Schulman J.L., Khakpour M., Kilbourne E.D. (1968). Protective effects of specific immunity to viral neuraminidase on influenza virus infection of mice. J. Virol..

[27] Wohlbold T.J., Nachbagauer R., Xu H., Tan G.S., Hirsh A., Brokstad K.A., Cox R.J., Palese P., Krammer F. (2015). Vaccination with adjuvanted recombinant neuraminidase induces broad heterologous, but not heterosubtypic, cross-protection against influenza virus infection in mice. mBio.

[28] Smith G.E., Sun X., Bai Y., Liu Y.V., Massare M.J., Pearce M.B., Belser J.A., Maines T.R., Creager H.M., Glenn G.M. (2017). Neuraminidase-based recombinant virus-like particles protect against lethal avian influenza A(H5N1) virus infection in ferrets. Virology.

[29] Couch R.B., Kasel J.A., Gerin J.L., Schulman J.L., Kilbourne E.D. (1974). Induction of partial immunity to influenza by a neuraminidase-specific vaccine. J. Infect. Dis..

[30] Murphy B.R., Kasel J.A., Chanock R.M. (1972). Association of serum anti-neuraminidase antibody with resistance to influenza in man. N. Engl. J. Med..

[31] Memoli M.J., Shaw P.A., Han A., Czajkowski L., Reed S., Athota R., Bristol T., Fargis S., Risos K., Powers J.H. (2016). Evaluation of Antihemagglutinin and Antineuraminidase Antibodies as Correlates of Protection in an Influenza A/H1N1 Virus Healthy Human Challenge Model. mBio.

[32] Maier H.E., Nachbagauer R., Kuan G., Ng S., Lopez R., Sanchez N., Stadlbauer D., Gresh L., Schiller A., Rajabhathor A. (2019). Pre-existing anti-neuraminidase antibodies are associated with shortened duration of influenza A (H1N1)pdm virus shedding and illness in naturally infected adults. Clin. Infect. Dis..

[33] Couch R.B., Atmar R.L., Franco L.M., Quarles J.M., Wells J., Arden N., Nino D., Belmont J.W. (2013). Antibody correlates and predictors of immunity to naturally occurring influenza in humans and the importance of antibody to the neuraminidase. J. Infect. Dis..

[34] Monto A.S., Petrie J.G., Cross R.T., Johnson E., Liu M., Zhong W., Levine M., Katz J.M., Ohmit S.E. (2015). Antibody to Influenza Virus Neuraminidase: An Independent Correlate of Protection. J. Infect. Dis..

[35] Smith W., Andrews C.H., Laidlaw P.P. (1933). A virus obtained from influenza patients. Lancet.

[36] Johansson B.E., Moran T.M., Bona C.A., Popple S.W., Kilbourne E.D. (1987). Immunologic response to influenza virus neuraminidase is influenced by prior experience with the associated viral hemagglutinin. II. Sequential infection of mice simulates human experience. J. Immunol..

[37] Chen Y.Q., Wohlbold T.J., Zheng N.Y., Huang M., Huang Y., Neu K.E., Lee J., Wan H., Rojas K.T., Kirkpatrick E. (2018). Influenza Infection in Humans Induces Broadly Cross-Reactive and Protective Neuraminidase-Reactive Antibodies. Cell.

[38] Nachbagauer R., Choi A., Hirsh A., Margine I., Iida S., Barrera A., Ferres M., Albrecht R.A., Garcia-Sastre A., Bouvier N.M. (2017). Defining the antibody cross-reactome directed against the influenza virus surface glycoproteins. Nat. Immunol..

[39] Changsom D., Jiang L., Lerdsamran H., Iamsirithaworn S., Kitphati R., Pooruk P., Auewarakul P., Puthavathana P. (2017). Kinetics, Longevity, and Cross-Reactivity of Antineuraminidase Antibody after Natural Infection with Influenza A Viruses. Clin. Vaccine Immunol..

[40] Smith A.J., Davies J.R. (1976). Natural infection with influenza A (H3N2). The development, persistance and effect of antibodies to the surface antigens. Epidemiol. Infect..

[41] Schild G.C. (1969). Antibody against influenza A2 virus neuraminidase in human sera. J. Hyg..

[42] Rajendran M., Nachbagauer R., Ermler M.E., Bunduc P., Amanat F., Izikson R., Cox M., Palese P., Eichelberger M., Krammer F. (2017). Analysis of Anti-Influenza Virus Neuraminidase Antibodies in Children, Adults, and the Elderly by ELISA and Enzyme Inhibition: Evidence for Original Antigenic Sin. mBio.

[43] Gao Z., Robinson K., Skowronski D.M., De Serres G., Withers S.G. (2020). Quantification of the total neuraminidase content of recent commercially-available influenza vaccines: Introducing a neuraminidase titration reagent. Vaccine.

[44] Petrie J.G., Ohmit S.E., Johnson E., Truscon R., Monto A.S. (2015). Persistence of Antibodies to Influenza Hemagglutinin and Neuraminidase Following One or Two Years of Influenza Vaccination. J. Infect. Dis..

[45] Ehrlich H.J., Muller M., Kollaritsch H., Pinl F., Schmitt B., Zeitlinger M., Loew-Baselli A., Kreil T.R., Kistner O., Portsmouth D. (2012). Pre-vaccination immunity and immune responses to a cell culture-derived whole-virus H1N1 vaccine are similar to a seasonal influenza vaccine. Vaccine.

[46] Couch R.B., Atmar R.L., Keitel W.A., Quarles J.M., Wells J., Arden N., Nino D. (2012). Randomized comparative study of the serum antihemagglutinin and antineuraminidase antibody responses to six licensed trivalent influenza vaccines. Vaccine.

[47] Laguio-Vila M.R., Thompson M.G., Reynolds S., Spencer S.M., Gaglani M., Naleway A., Ball S., Bozeman S., Baker S., Martinez-Sobrido L. (2015). Comparison of serum hemagglutinin and neuraminidase inhibition antibodies after 2010-2011 trivalent inactivated influenza vaccination in healthcare personnel. Open Forum Infect. Dis..

[48] Gross P.A., Russo C., Dran S., Cataruozolo P., Munk G., Lancey S.C. (1997). Time to earliest peak serum antibody response to influenza vaccine in the elderly. Clin. Diagn. Lab. Immunol..

[49] Gross P.A., Russo C., Teplitzky M., Dran S., Cataruozolo P., Munk G. (1996). Time to peak serum antibody response to influenza vaccine in the elderly. Clin. Diagn. Lab. Immunol..

[50] Rastogi S., Gross P.A., Bonelli J., Dran S., Levandowski R.A., Russo C., Weksler M.E., Kaye D., Levison M., Abrutyn E. (1995). Time to peak serum antibody response to influenza vaccine. Clin. Diagn. Lab. Immunol..

[51] Kositanont U., Assantachai P., Wasi C., Puthavathana P., Praditsuwan R. (2012). Kinetics of the antibody response to seasonal influenza vaccination among the elderly. Viral Immunol..

[52] Mohn K.G., Smith I., Sjursen H., Cox R.J. (2018). Immune responses after live attenuated influenza vaccination. Hum. Vaccin. Immunother..

[53] Ghendon Y. (1990). The immune response to influenza vaccines. Acta Virol..

[54] Belongia E.A., Sundaram M.E., McClure D.L., Meece J.K., Ferdinands J., VanWormer J.J. (2015). Waning vaccine protection against influenza A (H3N2) illness in children and older adults during a single season. Vaccine.

[55] Puig-Barbera J., Mira-Iglesias A., Tortajada-Girbes M., Lopez-Labrador F.X., Librero-Lopez J., Diez-Domingo J., Carballido-Fernandez M., Carratala-Munuera C., Correcher-Medina P., Gil-Guillen V. (2017). Waning protection of influenza vaccination during four influenza seasons, 2011/2012 to 2014/2015. Vaccine.

[56] Kirkpatrick E., Qiu X., Wilson P.C., Bahl J., Krammer F. (2018). The influenza virus hemagglutinin head evolves faster than the stalk domain. Sci. Rep..

[57] Bangaru S., Lang S., Schotsaert M., Vanderven H.A., Zhu X., Kose N., Bombardi R., Finn J.A., Kent S.J., Gilchuk P. (2019). A Site of Vulnerability on the Influenza Virus Hemagglutinin Head Domain Trimer Interface. Cell.

[58] Corti D., Cameroni E., Guarino B., Kallewaard N.L., Zhu Q., Lanzavecchia A. (2017). Tackling influenza with broadly neutralizing antibodies. Curr. Opin. Virol..

[59] Tay M.Z., Wiehe K., Pollara J. (2019). Antibody-Dependent Cellular Phagocytosis in Antiviral Immune Responses. Front. Immunol..

[60] Jegaskanda S. (2018). The Potential Role of Fc-Receptor Functions in the Development of a Universal Influenza Vaccine. Vaccines.

[61] Liu Y., Tan H.X., Koutsakos M., Jegaskanda S., Esterbauer R., Tilmanis D., Aban M., Kedzierska K., Hurt A.C., Kent S.J. (2019). Cross-lineage protection by human antibodies binding the influenza B hemagglutinin. Nat. Commun..

[62] Whittle J.R., Zhang R., Khurana S., King L.R., Manischewitz J., Golding H., Dormitzer P.R., Haynes B.F., Walter E.B., Moody M.A. (2011). Broadly neutralizing human antibody that recognizes the receptor-binding pocket of influenza virus hemagglutinin. Proc. Natl. Acad. Sci. USA.

[63] Zost S.J., Lee J., Gumina M.E., Parkhouse K., Henry C., Wu N.C., Lee C.D., Wilson I.A., Wilson P.C., Bloom J.D. (2019). Identification of Antibodies Targeting the H3N2 Hemagglutinin Receptor Binding Site following Vaccination of Humans. Cell.

[64] Shen C., Zhang M., Chen Y., Zhang L., Wang G., Chen J., Chen S., Li Z., Wei F., Chen J. (2019). An IgM antibody targeting the receptor binding site of influenza B blocks viral infection with great breadth and potency. Theranostics.

[65] Krammer F., Palese P. (2013). Influenza virus hemagglutinin stalk-based antibodies and vaccines. Curr. Opin. Virol..

[66] Rajendran M., Sun W., Comella P., Nachbagauer R., Wohlbold T.J., Amanat F., Kirkpatrick E., Palese P., Krammer F. (2018). An immuno-assay to quantify influenza virus hemagglutinin with correctly folded stalk domains in vaccine preparations. PLoS ONE.

[67] Sui J., Hwang W.C., Perez S., Wei G., Aird D., Chen L.M., Santelli E., Stec B., Cadwell G., Ali M. (2009). Structural and functional bases for broad-spectrum neutralization of avian and human influenza A viruses. Nat. Struct. Mol. Biol..

[68] Ekiert D.C., Bhabha G., Elsliger M.A., Friesen R.H., Jongeneelen M., Throsby M., Goudsmit J., Wilson I.A. (2009). Antibody recognition of a highly conserved influenza virus epitope. Science.

[69] Ekiert D.C., Friesen R.H., Bhabha G., Kwaks T., Jongeneelen M., Yu W., Ophorst C., Cox F., Korse H.J., Brandenburg B. (2011). A highly conserved neutralizing epitope on group 2 influenza A viruses. Science.

[70] Gilchuk I.M., Bangaru S., Gilchuk P., Irving R.P., Kose N., Bombardi R.G., Thornburg N.J., Creech C.B., Edwards K.M., Li S. (2019). Influenza H7N9 Virus Neuraminidase-Specific Human Monoclonal Antibodies Inhibit Viral Egress and Protect from Lethal Influenza Infection in Mice. Cell Host Microbe.

[71] Jiang L., Fantoni G., Couzens L., Gao J., Plant E., Ye Z., Eichelberger M.C., Wan H. (2016). Comparative Efficacy of Monoclonal Antibodies That Bind to Different Epitopes of the 2009 Pandemic H1N1 Influenza Virus Neuraminidase. J. Virol..

[72] Wan H., Gao J., Xu K., Chen H., Couzens L.K., Rivers K.H., Easterbrook J.D., Yang K., Zhong L., Rajabi M. (2013). Molecular basis for broad neuraminidase immunity: Conserved epitopes in seasonal and pandemic H1N1 as well as H5N1 influenza viruses. J. Virol..

[73] Wohlbold T.J., Chromikova V., Tan G.S., Meade P., Amanat F., Comella P., Hirsh A., Krammer F. (2016). Hemagglutinin Stalk- and Neuraminidase-Specific Monoclonal Antibodies Protect against Lethal H10N8 Influenza Virus Infection in Mice. J. Virol..

[74] Shcherbik S., Carney P., Pearce N., Stevens J., Dugan V.G., Wentworth D.E., Bousse T. (2018). Monoclonal antibody against N2 neuraminidase of cold adapted A/Leningrad/134/17/57 (H2N2) enables efficient generation of live attenuated influenza vaccines. Virology.

[75] Job E.R., Ysenbaert T., Smet A., Van Hecke A., Meuris L., Kleanthous H., Saelens X., Vogel T.U. (2019). Fcgamma Receptors Contribute to the Antiviral Properties of Influenza Virus Neuraminidase-Specific Antibodies. mBio.

[76] Von Holle T.A., Moody M.A. (2019). Influenza and Antibody-Dependent Cellular Cytotoxicity. Front. Immunol..

[77] Jegaskanda S., Reading P.C., Kent S.J. (2014). Influenza-specific antibody-dependent cellular cytotoxicity: Toward a universal influenza vaccine. J. Immunol..

[78] Jegaskanda S., Weinfurter J.T., Friedrich T.C., Kent S.J. (2013). Antibody-dependent cellular cytotoxicity is associated with control of pandemic H1N1 influenza virus infection of macaques. J. Virol..

[79] Valkenburg S.A., Fang V.J., Leung N.H., Chu D.K., Ip D.K., Perera R.A., Wang Y., Li A.P., Peiris J.M., Cowling B.J. (2019). Cross-reactive antibody-dependent cellular cytotoxicity antibodies are increased by recent infection in a household study of influenza transmission. Clin. Transl. Immunol..

[80] Rijal P., Wang B.B., Tan T.K., Schimanski L., Janesch P., Dong T., McCauley J.W., Daniels R.S., Townsend A.R., Huang K.A. (2020). Broadly Inhibiting Antineuraminidase Monoclonal Antibodies Induced by Trivalent Influenza Vaccine and H7N9 Infection in Humans. J. Virol..

[81] Zhu X., Turner H.L., Lang S., McBride R., Bangaru S., Gilchuk I.M., Yu W., Paulson J.C., Crowe J.E., Ward A.B. (2019). Structural Basis of Protection against H7N9 Influenza Virus by Human Anti-N9 Neuraminidase Antibodies. Cell Host Microbe.

[82] Krammer F., Li L., Wilson P.C. (2019). Emerging from the Shadow of Hemagglutinin: Neuraminidase Is an Important Target for Influenza Vaccination. Cell Host Microbe.

[83] Madsen A., Dai Y.N., McMahon M., Schmitz A.J., Turner J.S., Tan J., Lei T., Alsoussi W.B., Strohmeier S., Amor M. (2020). Human Antibodies Targeting Influenza B Virus Neuraminidase Active Site Are Broadly Protective. Immunity.

[84] Piepenbrink M.S., Nogales A., Basu M., Fucile C.F., Liesveld J.L., Keefer M.C., Rosenberg A.F., Martinez-Sobrido L., Kobie J.J. (2019). Broad and Protective Influenza B Virus Neuraminidase Antibodies in Humans after Vaccination and their Clonal Persistence as Plasma Cells. mBio.

[85] Stadlbauer D., Zhu X., McMahon M., Turner J.S., Wohlbold T.J., Schmitz A.J., Strohmeier S., Yu W., Nachbagauer R., Mudd P.A. (2019). Broadly protective human antibodies that target the active site of influenza virus neuraminidase. Science.

[86] Arunkumar G.A., Strohmeier S., Li T., Bhavsar D., Chromikova V., Amanat F., Bunyatov M., Wilson P.C., Ellebedy A.H., Boons G.-J. (1968). Reactions of antibodies with surface antigens of influenza virus. J. Gen. Virol..

[87] Webster R.G., Laver W.G., Kilbourne E.D. (1968). Reactions of Antibodies with Surface Antigens of Influenza Virus. J. Gen. Virol..

[88] Westgeest K.B., Russell C.A., Lin X., Spronken M.I., Bestebroer T.M., Bahl J., van Beek R., Skepner E., Halpin R.A., de Jong J.C. (2014). Genomewide analysis of reassortment and evolution of human influenza A(H3N2) viruses circulating between 1968 and 2011. J. Virol..

[89] Hensley S.E., Das S.R., Gibbs J.S., Bailey A.L., Schmidt L.M., Bennink J.R., Yewdell J.W. (2011). Influenza A virus hemagglutinin antibody escape promotes neuraminidase antigenic variation and drug resistance. PLoS ONE.

[90] Johansson B.E., Bucher D.J., Kilbourne E.D. (1989). Purified influenza virus hemagglutinin and neuraminidase are equivalent in stimulation of antibody response but induce contrasting types of immunity to infection. J. Virol..

[91] Matthew J., Syle D.L.S., Richard W., Compans W.A.O. (2009). Influenza Neuraminidase as a Vaccine Antigen. Vaccines for Pandemic Influenza.

[92] Jagadesh A., Salam A.A., Mudgal P.P., Arunkumar G. (2016). Influenza virus neuraminidase (NA): A target for antivirals and vaccines. Arch. Virol..

[93] Getie-Kebtie M., Sultana I., Eichelberger M., Alterman M. (2013). Label-free mass spectrometry-based quantification of hemagglutinin and neuraminidase in influenza virus preparations and vaccines. Influenza Other Respir. Viruses.

[94] Williams T.L., Pirkle J.L., Barr J.R. (2012). Simultaneous quantification of hemagglutinin and neuraminidase of influenza virus using isotope dilution mass spectrometry. Vaccine.

[95] Wan H., Sultana I., Couzens L.K., Mindaye S., Eichelberger M.C. (2017). Assessment of influenza A neuraminidase (subtype N1) potency by ELISA. J. Virol. Methods.

[96] Broecker F., Zheng A., Suntronwong N., Sun W., Bailey M.J., Krammer F., Palese P. (2019). Extending the Stalk Enhances Immunogenicity of the Influenza Virus Neuraminidase. J. Virol..

[97] Kilbourne E.D., Couch R.B., Kasel J.A., Keitel W.A., Cate T.R., Quarles J.H., Grajower B., Pokorny B.A., Johansson B.E. (1995). Purified influenza A virus N2 neuraminidase vaccine is immunogenic and non-toxic in humans. Vaccine.

[98] Liu W.C., Lin C.Y., Tsou Y.T., Jan J.T., Wu S.C. (2015). Cross-Reactive Neuraminidase-Inhibiting Antibodies Elicited by Immunization with Recombinant Neuraminidase Proteins of H5N1 and Pandemic H1N1 Influenza A Viruses. J. Virol..

[99] Job E.R., Ysenbaert T., Smet A., Christopoulou I., Strugnell T., Oloo E.O., Oomen R.P., Kleanthous H., Vogel T.U., Saelens X. (2018). Broadened immunity against influenza by vaccination with computationally designed influenza virus N1 neuraminidase constructs. NPJ Vaccines.

[100] Brett I.C., Johansson B.E. (2005). Immunization against influenza A virus: Comparison of conventional inactivated, live-attenuated and recombinant baculovirus produced purified hemagglutinin and neuraminidase vaccines in a murine model system. Virology.

[101] Johansson B.E., Brett I.C. (2008). Recombinant influenza B virus HA and NA antigens administered in equivalent amounts are immunogenically equivalent and induce equivalent homotypic and broader heterovariant protection in mice than conventional and live influenza vaccines. Hum. Vaccin..

[102] McMahon M., Kirkpatrick E., Stadlbauer D., Strohmeier S., Bouvier N.M., Krammer F. (2019). Mucosal Immunity against Neuraminidase Prevents Influenza B Virus Transmission in Guinea Pigs. mBio.

[103] Kim K.H., Lee Y.T., Park S., Jung Y.J., Lee Y., Ko E.J., Kim Y.J., Li X., Kang S.M. (2019). Neuraminidase expressing virus-like particle vaccine provides effective cross protection against influenza virus. Virology.

[104] Easterbrook J.D., Schwartzman L.M., Gao J., Kash J.C., Morens D.M., Couzens L., Wan H., Eichelberger M.C., Taubenberger J.K. (2012). Protection against a lethal H5N1 influenza challenge by intranasal immunization with virus-like particles containing 2009 pandemic H1N1 neuraminidase in mice. Virology.

[105] Halbherr S.J., Ludersdorfer T.H., Ricklin M., Locher S., Berger Rentsch M., Summerfield A., Zimmer G. (2015). Biological and protective properties of immune sera directed to the influenza virus neuraminidase. J. Virol..

[106] Mooney A.J., Gabbard J.D., Li Z., Dlugolenski D.A., Johnson S.K., Tripp R.A., He B., Tompkins S.M. (2017). Vaccination with Recombinant Parainfluenza Virus 5 Expressing Neuraminidase Protects against Homologous and Heterologous Influenza Virus Challenge. J. Virol..

[107] Meseda C.A., Atukorale V., Soto J., Eichelberger M.C., Gao J., Wang W., Weiss C.D., Weir J.P. (2018). Immunogenicity and Protection Against Influenza H7N3 in Mice by Modified Vaccinia Virus Ankara Vectors Expressing Influenza Virus Hemagglutinin or Neuraminidase. Sci. Rep..

[108] Sandbulte M.R., Jimenez G.S., Boon A.C., Smith L.R., Treanor J.J., Webby R.J. (2007). Cross-reactive neuraminidase antibodies afford partial protection against H5N1 in mice and are present in unexposed humans. PLoS Med..

[109] Chen Z., Kadowaki S., Hagiwara Y., Yoshikawa T., Matsuo K., Kurata T., Tamura S. (2000). Cross-protection against a lethal influenza virus infection by DNA vaccine to neuraminidase. Vaccine.

[110] Freyn A.W., Ramos da Silva J., Rosado V.C., Bliss C.M., Pine M., Mui B.L., Tam Y.K., Madden T.D., de Souza Ferreira L.C., Weissman D. (2020). A Multi-Targeting, Nucleoside-Modified mRNA Influenza Virus Vaccine Provides Broad Protection in Mice. Mol. Ther..

[111] Nicholson K.G., Webster R.G., Hay A., Woods J.M. (1998). Standardization of Inactivated Influenza Vaccines. Textbook of Influenza.

[112] Van Deusen R.A., Hinshaw V.S., Senne D.A., Pellacani D. (1983). Micro neuraminidase-inhibition assay for classification of influenza A virus neuraminidases. Avian Dis..

[113] Aminoff D. (1961). Methods for the quantitative estimation of N-acetylneuraminic acid and their application to hydrolysates of sialomucoids. Biochem. J..

[114] Eichelberger M.C., Monto A.S. (2019). Neuraminidase, the Forgotten Surface Antigen, Emerges as an Influenza Vaccine Target for Broadened Protection. J. Infect. Dis..

[115] Gomez Lorenzo M.M., Fenton M.J. (2013). Immunobiology of influenza vaccines. Chest.

[116] Sultana I., Yang K., Getie-Kebtie M., Couzens L., Markoff L., Alterman M., Eichelberger M.C. (2014). Stability of neuraminidase in inactivated influenza vaccines. Vaccine.

[117] Zheng A., Sun W., Xiong X., Freyn A.W., Peukes J., Strohmeier S., Nachbagauer R., Briggs J.A.G., Krammer F., Palese P. (2020). Enhancing Neuraminidase Immunogenicity of Influenza A Viruses by Rewiring RNA Packaging Signals. J. Virol..

[118] Roldao A., Mellado M.C., Castilho L.R., Carrondo M.J., Alves P.M. (2010). Virus-like particles in vaccine development. Expert Rev. Vaccines.

[119] Jegerlehner A., Zabel F., Langer A., Dietmeier K., Jennings G.T., Saudan P., Bachmann M.F. (2013). Bacterially produced recombinant influenza vaccines based on virus-like particles. PLoS ONE.

[120] Zhao Q., Li S., Yu H., Xia N., Modis Y. (2013). Virus-like particle-based human vaccines: Quality assessment based on structural and functional properties. Trends Biotechnol..

[121] Lundstrom K. (2016). Replicon RNA Viral Vectors as Vaccines. Vaccines.

[122] Zimmer G. (2010). RNA replicons—A new approach for influenza virus immunoprophylaxis. Viruses.

[123] Hoelscher M.A., Garg S., Bangari D.S., Belser J.A., Lu X., Stephenson I., Bright R.A., Katz J.M., Mittal S.K., Sambhara S. (2006). Development of adenoviral-vector-based pandemic influenza vaccine against antigenically distinct human H5N1 strains in mice. Lancet.

[124] Yang S.G., Wo J.E., Li M.W., Mi F.F., Yu C.B., Lv G.L., Cao H.C., Lu H.F., Wang B.H., Zhu H. (2009). Construction and cellular immune response induction of HA-based alphavirus replicon vaccines against human-avian influenza (H5N1). Vaccine.

[125] Choi Y., Chang J. (2013). Viral vectors for vaccine applications. Clin. Exp. Vaccine Res..

[126] Kim J.H., Jacob J. (2009). DNA vaccines against influenza viruses. Curr. Top. Microbiol. Immunol..

[127] Wolff J.A., Malone R.W., Williams P., Chong W., Acsadi G., Jani A., Felgner P.L. (1990). Direct gene transfer into mouse muscle in vivo. Science.

[128] Ulmer J.B., Mason P.W., Geall A., Mandl C.W. (2012). RNA-based vaccines. Vaccine.

[129] Rodriguez-Gascon A., del Pozo-Rodriguez A., Solinis M.A. (2014). Development of nucleic acid vaccines: Use of self-amplifying RNA in lipid nanoparticles. Int. J. Nanomed..

